# The self-reported Montgomery-Åsberg depression rating scale is a useful evaluative tool in major depressive disorder

**DOI:** 10.1186/1471-244X-9-26

**Published:** 2009-05-27

**Authors:** Bruno Fantino, Nicholas Moore

**Affiliations:** 1 ADIM-AGORAS, 112, cours Albert Thomas, 69008 LYON, France; 2 Département de Pharmacologie, CHU de Bordeaux – Université Victor Segalen, Case 36, 33076 Bordeaux, France

## Abstract

**Background:**

The use of Patient-reported Outcomes (PROs) as secondary endpoints in the development of new antidepressants has grown in recent years. The objective of this study was to assess the psychometric properties of the 9-item, patient-administered version of the Montgomery-Åsberg Depression Rating Scale (MADRS-S).

**Methods:**

Data from a multicentre, double-blind, 8-week, randomised controlled trial of 278 outpatients diagnosed with Major Depressive Disorder were used to evaluate the validity, reliability and sensitivity to change of the MADRS-S using psychometric methods. A Receiver Operating Characteristic (ROC) curve was plotted to identify the most appropriate threshold to define perceived remission.

**Results:**

No missing values were found at the item level, indicating good acceptability of the scale. The construct validity was satisfactory: all items contributed to a common underlying concept, as expected. The correlation between MADRS-S and physicians' MADRS was moderate (r = 0.54, p < 0.001) indicating that MADRS-S is complementary rather than redundant to the MADRS. Cronbach's alpha was 0.84, and the stability over time of the scale, estimated on a sub-sample of patients whose health status did not change during the first week of the study, was good (intraclass correlation coefficient of 0.78). MADRS-S sensitivity to change was shown. Using a threshold value of 5, the definition of "perceived remission" reached a sensitivity of 82% and a specificity of 75%.

**Conclusion:**

Taking account of patient's perceptions of the severity of their own symptoms along with the psychometric properties of the MADRS-S enable its use for evaluative purposes in the development of new antidepressant drugs.

## Introduction

Depression is a disabling illness associated with considerable co-morbidity, risk of suicide and numerous adverse social and economic consequences [1-3]. The reported lifetime risk for Major Depressive Disorder (MDD) in the general population varies between 10% – 25% for women, and 5% – 12% for men [4]. The pharmacological treatment of MDD is based on antidepressants, whose efficacy has been demonstrated in a large number of studies [5].

The use of patient-reported outcomes as a secondary endpoint in the development of new antidepressants has been of growing interest in recent years. Among these outcomes, health-related quality of life, medication compliance and subjective effectiveness (patients' perceptions of symptom severity) are the most commonly used [6-8]. One possible explanation for this growing interest is the fact that patients are increasingly becoming key players in the overall disease management process. In the subjective effectiveness questionnaire category, the main instruments are the Beck Depression Inventory (BDI) [9,10], the Carroll Rating Scale for Depression (CRSD) [11-13], the Montgomery – Åsberg Depression Rating Scale – Self report (MADRS-S) [14], the Hamilton Depression Inventory (HDI) [15], and the Quick Inventory of Depressive Symptomatology – Self Report (QIDS-SR) [16].

The BDI is the most widely used self-rating instrument, and has been extensively validated in numerous studies [17]. The CRSD and the HDI are the self-reported versions of the Hamilton Depression Rating Scale (HDRS) [18], while the MADRS-S is the patient version of the Montgomery-Åsberg Depression Rating Scale (MADRS) [19]. From the conceptual and psychometric points of view, these questionnaires are quite different. The BDI is more concerned with depressive cognitive attitudes while the other scales pay more attention to somatic symptoms and functional impairment. The CRSD may discourage patients; completing its 52 questions is time consuming. This may also make it more difficult to implement in clinical research studies compared with the shorter 16-item QIDS-SR or 9-item MADRS-S.

These reasons led us to focus on the psychometric properties of the MADRS-S, particularly its sensitivity to change, since this is of major importance for evaluative purposes (e.g. in the comparison of treatment effects).

## Patients and Methods

### Study Design and Population

Data came from a multicentre, double blind, randomised clinical trial comparing escitalopram with citalopram in outpatients diagnosed with MDD [20]. Eligible patients were aged between 18–65 years, fulfilled DSM-IV criteria for MDD, and had a baseline MADRS total score of at least 30.

Patients meeting DSM-IV criteria for primary diagnoses of any axis I other than MDD, or those with a history of mania, bipolar disorder, schizophrenia or other psychotic disorder, obsessive-compulsive disorder, or cognitive disorder were not eligible for the study. Patients who met DSM-IV criteria for substance abuse or dependence within the past 12 moths, or who used a depot antipsychotic within 6 months before study inclusion, or any antipsychotic, anxiolytic or anticonvulsant medications within 2 weeks before the first administration of study medication were also ineligible for inclusion.

The Regional review and Ethics committee approved the study protocol on September 3^rd^, 2003. All patients provided their written informed consent.

### Assessments

Study assessments were performed at baseline and at weeks 1, 4 and 8 after start of treatment. Sociodemographics and clinical data were collected at baseline, and the investigators administered the MADRS, the Clinical Global Impression of Severity (CGI-S) and Improvement (CGI-I) scales at each visit [21]. Before these assessments, patients were asked to fill in the MADRS-S.

This scale consists of 9 items assessing patients' mood, feelings of unease, sleep, appetite, ability to concentrate, initiative, emotional involvement, pessimism and zest for life. Each item is scored between 0 and 3, with three intermediate levels (0.5, 1.5, 2.5). The total score is calculated by summing the answers of the nine items, ranging between 0 and 27 (higher scores indicate increased impairment).

### Statistical analyses

Statistical analyses were performed using SAS version 8.2 [22], and all statistical tests were two-sided. The α risk was set to 0.05. Continuous variables were described using mean ± standard deviation (SD), while categorical variables were reported using frequency and percentage.

Item-level analysis consisted of assessing the number of missing values for each item and item-response distribution [23]. Correlating each item with the MADRS-S total score after correction for overlap assessed item-internal consistency. A correlation of at least 0.40 is recommended as the standard for supporting item-internal consistency [24]. We also calculated the percentage of respondents achieving the lowest (floor effect) and highest (ceiling effect) score to determine whether the range of MADRS-S was appropriate.

Construct validity was examined using several methods.

(1) Factor analysis was conducted to test the underlying dimensionality of the MADRS-S.

(2) The discriminative validity of the MADRS-S was determined by comparing mean scores across patient groups that were known to differ in their clinical features (known-groups methodology [25]). Since the recall period of the MADRS-S is the past three days, we did not expect it to be associated with medical history (i.e. number of episodes of depression, history of psychiatric hospitalisation), and we assumed the MADRS-S total score to be associated with the severity of the current episode.

(3) The Receiver Operating Characteristic (ROC) curve was plotted to define the optimal cut-off value for perceived remission, using the MADRS criteria of 12 or less for remission as the "gold standard".

Cronbach's alpha coefficient was used to estimate the internal consistency reliability of the MADRS-S score. A reliability of at least 0.70 is recommended to compare groups of patients, while at least 0.90 is required for comparing individuals [26]. Test-retest reliability of the MADRS-S questionnaire was assessed in a sub-sample of 120 patients whose health status severity was declared unchanged between the baseline and week 1 visits using the CGI-I scale. The intraclass correlation coefficient (ICC) was computed between scale scores from both assessments.

The sensitivity to change of the MADRS-S questionnaire was assessed in a sub-sample of 132 patients whose MADRS total score at week 8 was lower or equal to 12 (remission state). Baseline and week 8 scores were compared using paired *t*-test. Effect sizes were also computed. According to Cohen [27], an effect size of at least 0.2 is recommended as the standard for supporting sensitivity to change.

Finally, the evaluative ability of the MADRS-S to discriminate between treatment groups was tested using an analysis of covariance (ANCOVA) model, predicting the mean MADRS-S change at week 8 from baseline, with investigator specialisation and treatment as factors, and baseline MADRS-S score as covariate. Perceived response rates, defined as a reduction of at least 50% from the baseline MADRS-S score at week 8, and perceived remission rates, defined using the cut-off value revealed in the ROC analysis, were also compared using a logistic model with the same explanatory factors and covariate as those used in the ANCOVA model described above.

## Results

### Sample characteristics

Among the 280 patients, two (0.7%) refused to fill in the MADRS-S questionnaire and were excluded from the analyses. The mean patient age was 45.2 ± 11.0 years, 186 (66.9%) were females, 188 (67.6%) had a professional activity, and 218 (78.4%) lived in an urban area. One hundred and sixty-seven patients (60.1%) were recruited by psychiatrists; 137 patients (49.3%) were treated with escitalopram and the remaining 141 (50.7%) received citalopram.

Clinical characteristics are presented in Table 1. More than half of the patients were experiencing their first episode of MDD, while 46 (16.5%) had a history of psychiatric hospitalisation. Overall, patients had a mean MADRS of 35.9 and a mean CGI-S of 5.1; 57.6% were rated as severely ill (MADRS ≥ 35).

**Table 1:** Patients' clinical characteristics at baseline.

	**Overall Population (n = 278)**
**Age at first diagnosis of MDD**, mean ± SD	39.5 ± 12.0

**First episode of MDD**, n (%)	
Yes	153 (55.0%)
No	125 (45.0%)

**History of psychiatric hospitalisation**, n (%)	
Yes	46 (16.5%)
No	232 (83.5%)

**Clinical Global Impression of Severity**, mean ± SD	5.1 ± 0.5

**MADRS Total Score**, mean ± SD	35.9 ± 4.5

**MADRS Total Score ≥ 35**, n (%)	
Yes	160 (57.6%)
No	118 (42.4%)

### Item-level analysis

No missing values were observed, indicating a high level of patient acceptability of the questionnaire. With the exception of items 4 (appetite), 7 (emotional involvement) and 9 (zest for life), item response distributions had a higher ceiling rather than floor effect, highlighting the initial severity of the disease (Table 2). Item-scale correlations showed that all but one item (8, pessimism) achieved the standard value of 0.40 for item-internal consistency (Table 2).

**Table 2:** Descriptive Statistics of the 9 MADRS-S Items and Total Score.

	**Missing Values**	**Mean ± SD**	**Correlation^a^**	**Cronbach's α**	**% Floor**	**%Ceiling**
**Item 1: Mood**	0	1.86 ± 0.84	0.68 ***	0.80^b^	10.1%	12.9%
**Item 2: Feelings of Unease**	0	2.06 ± 0.63	0.61 ***	0.82^b^	2.2%	15.5%
**Item 3: Sleep**	0	1.94 ± 0.68	0.43 ***	0.83^b^	4.3%	10.1%
**Item 4: Appetite**	0	1.39 ± 0.93	0.43 ***	0.84^b^	21.6%	7.6%
**Item 5: Ability to Concentrate**	0	1.92 ± 0.66	0.64 ***	0.81^b^	2.5%	10.4%
**Item 6: Initiative**	0	1.91 ± 0.70	0.62 ***	0.81^b^	4.0%	7.2%
**Item 7: Emotional Involvement**	0	1.58 ± 0.75	0.65 ***	0.81^b^	7.6%	5.4%
**Item 8: Pessimism**	0	2.06 ± 0.72	0.39 ***	0.84^b^	1.1%	22.7%
**Item 9: Zest for Life**	0	1.44 ± 0.75	0.55 ***	0.82^b^	8.3%	4.7%

**MADRS-S Total Score**	0	16.2 ± 4.4	--	0.84^c^	0.0%	0.4%

### Construct validity

The results of the factor analysis confirmed the unidimensionality of the MADRS-S: each item contributed to the first factor axis with a factor loading of at least 0.50, explaining 45% of the total variance.

The MADRS-S score was moderately correlated with physicians' severity ratings (MADRS-S with MADRS: r = 0.54, p < 0.001; MADRS-S with CGI-S: r = 0.38, p < 0.001). As expected, the MADRS-S total score did not discriminate as to whether a patient was suffering from their first episode of MDD, nor if they had a history of psychiatric hospitalisation, but did discriminate as to whether a patient's baseline severity was ≥ 35 in the current episode (Table 3).

**Table 3:** Clinical Discriminative Validity of the MADRS-S.

	**Mean ± SD**	***p*-value**
**First episode of MDD**		
Yes (n = 153)	16.2 ± 4.4	0.87
No (n = 125)	16.1 ± 4.5	
**History of psychiatric hospitalisation**		
Yes (n = 46)	16.1 ± 5.1	0.92
No (n = 232)	16.2 ± 4.3	
**Severity of the current episode: Baseline MADRS ≥ 35**		
Yes (n = 160)	17.8 ± 4.0	< 0.001
No (n = 118)	14.0 ± 4.0	

**Figure 1:**
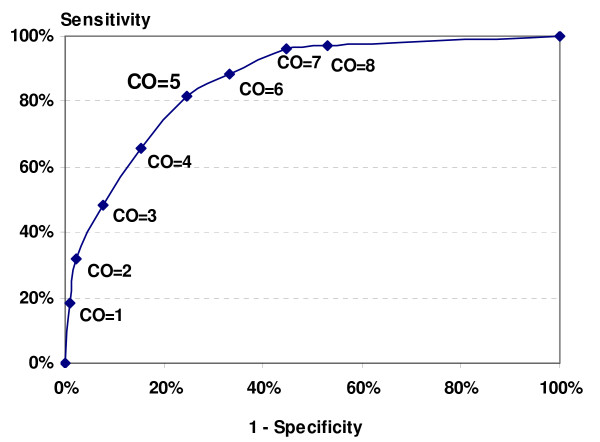
**Receiver Operating Characteristic Curve for Perceived Remission**. CO: cut-off value for MADRS-S score.

The ROC curve for perceived remission is displayed in Figure 1. Using the cut-off value of 5, the MADRS-S-based definition of perceived remission reached a sensitivity of 81.8%, a specificity of 75.4%, and positive and negative predicted values of 77.1% and 80.3%, respectively.

### Reliability

Internal consistency reliability of the MADRS-S was satisfactory, with a Cronbach's alpha of 0.84, allowing group comparisons. The deletion of any of the 9 items would not increase the internal consistency of the total score (Table 2).

Among the 120 patients whose CGI-I at week 1 was rated "No change" by physicians, the intraclass correlation coefficient was 0.78, indicating the satisfactory test-retest reliability of the MADRS-S.

### Sensitivity to changes

In the sub-sample of 132 remitter patients (i.e. those whose MADRS total score at week 8 was less than or equal to 12), a statistically significant difference of -12.4 ± 4.2 points was found for the total MADRS-S between baseline and week 8. This difference led to an effect size of 2.8, which supported the sensitivity to change of the self-reported version of the MADRS.

### Evaluative ability

When comparing the antidepressant effects of the two therapeutic strategies of the trial, we found that the mean MADRS-S score changes from baseline were in favour of escitalopram (-9.9 ± 5.1 for escitalopram *versus *-8.6 ± 5.9 for citalopram), the mean difference of 1.3 (standard error of 0.7) being statistically significant (*p *= 0.046). As a comparison, a mean MADRS difference of 2.1 was found between escitalopram and citalopram (p < 0.05).

**Figure 2:**
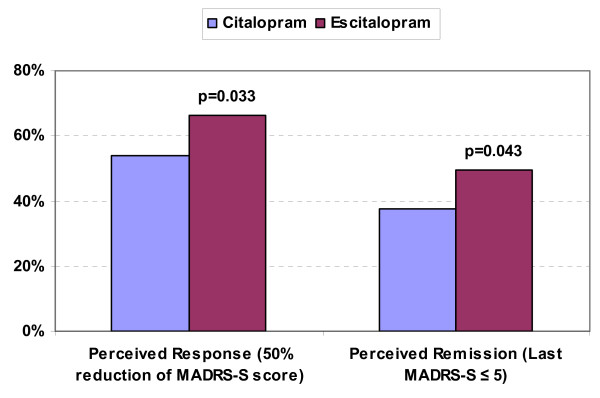
**Perceived Response and Perceived Remission at Week 8**.

Perceived response, defined as a reduction of at least 50% of the baseline MADRS-S score, and perceived remission, defined using the optimal cut-off value of 5 found in the ROC analysis, were also significantly in favour of escitalopram (Figure 2). Perceived response rates were 66.4% and 53.9% for escitalopram and citalopram, respectively (*p *= 0.033). Perceived remission rates were 49.6% and 37.6% for escitalopram and citalopram, respectively (*p *= 0.043). As a comparison, response rates based on investigators' ratings of the MADRS were 76.1% for escitalopram and 61.5% for citalopram (p = 0.009); remission rates were 56.1% and 43.6% for escitalopram and citalopram, respectively (p = 0.040).

## Discussion/Conclusion

The objective of this article was to investigate the psychometric properties of the MADRS-S, the patient-reported version of the MADRS. We demonstrated the validity, acceptability, reliability and sensitivity to change of the MADRS-S.

The lack of missing values illustrates good patient acceptance of the questionnaire, and indicates that it seems feasible to ask patients to rate their perception of nine symptoms of depression. Ceiling effects for six items were higher than floor effects, reflecting the initial severity level of the disease, as patients were only included in the study if they had a physician-reported MADRS score of at least 30. Results of the factor analysis supported the unique underlying concept assessed by the nine items. The reliability of the MADRS-S was satisfactory, with a test-retest intraclass correlation coefficient of 0.78 and a Cronbach's alpha of 0.84. Most importantly, the effect size of 2.8 in a sub-sample of improved patients after eight weeks of antidepressant treatment confirmed the ability of the scale to be sensitive to change, as is the original MADRS [19]. These results clearly showed the ability of the patient-reported MADRS-S to detect differences between treatment regimens.

However, the association between physician and patient-reported scores was lower in our study (0.54) compared with those reported by Svanborg and Åsberg (0.70) [14], and by Mundt *et al *(0.82) [28]. By comparison, Carroll *et al *[11] reported an association between the HDRS and the CRSD of 0.71. The more similar the scale, the higher the level of correlation found for assessment procedures (Mundt and colleagues compared the traditional clinician-rated MADRS with a telephone-based, interactive voice response technology); paper-and-pencil *vs*. interview-based ratings are known to only moderately correlate [29] and scales with slightly differing content and wording can be expected to show slightly lower correlations.

Patients were asked to fill the MADRS-S before any clinical assessments in order to provide the most accurate perception they have on their disease. It is noteworthy that response and remission rates based on the MADRS-S are always lower than those from the clinician-based MADRS, indicating that patients' and clinicians' perceptions of the disease are different, more complementary than redundant, and can provide additional useful information. This is of major interest for MDD management since taking into account patients' feelings may improve medication compliance, decrease time to symptom alleviation, and, as a result, improve patients' quality of life.

The growing focus on patient-reported outcomes [30] as a secondary endpoint in randomised clinical trials and the findings of our study lead us to recommend the concomitant use of the MADRS and MADRS-S during the development of new compounds.

## Competing interests

The authors declare that they have no competing interests.

## Authors' contributions

NM was head investigator and responsible for the design of the multicentre, double-blind, 8-week, randomised controlled trial of 278 outpatients from which data from the current study was provided. BF and NM conceived and participated in the design of the current analysis. BF oversaw execution of statistical analyses and was responsible for their coordination. Both authors read and approved the final manuscript.

## Pre-publication history

The pre-publication history for this paper can be accessed here:


